# Supplementary material for: Whence the next pandemic? The intersecting global geography of the animal-human interface, poor health systems and air transit centrality reveals conduits for high-impact spillover
Source: One Health. 2020 Oct 8;11:100177. doi: 10.1016/j.onehlt.2020.100177 (PMC7543735; doi:10.1016/j.onehlt.2020.100177)

S1 Figure 1. The global quartile distribution of mammalian and bird species richness, domesticated animals, human population, and infant mortality ratio (left column) and the corresponding 75^th^ percentile of each distribution (right column).


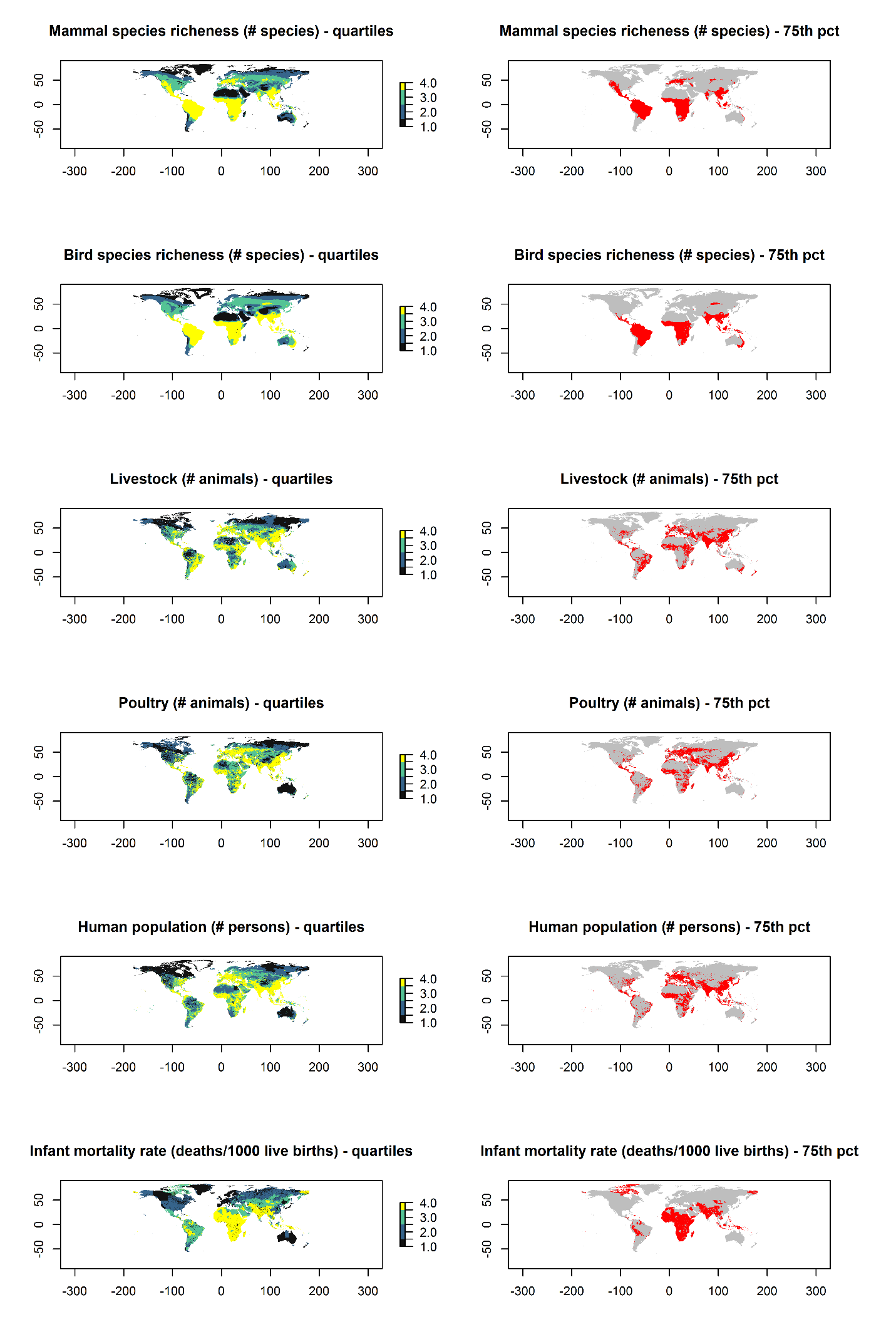


S2 Figure 2. The airport network graph using a large connected graph layout. Nodes represent airports with labels designating the International Air Transport Association airport codes and size reflecting the degree of airport centrality (eigenvector centrality). Edges represent connections by way of flights between airports.


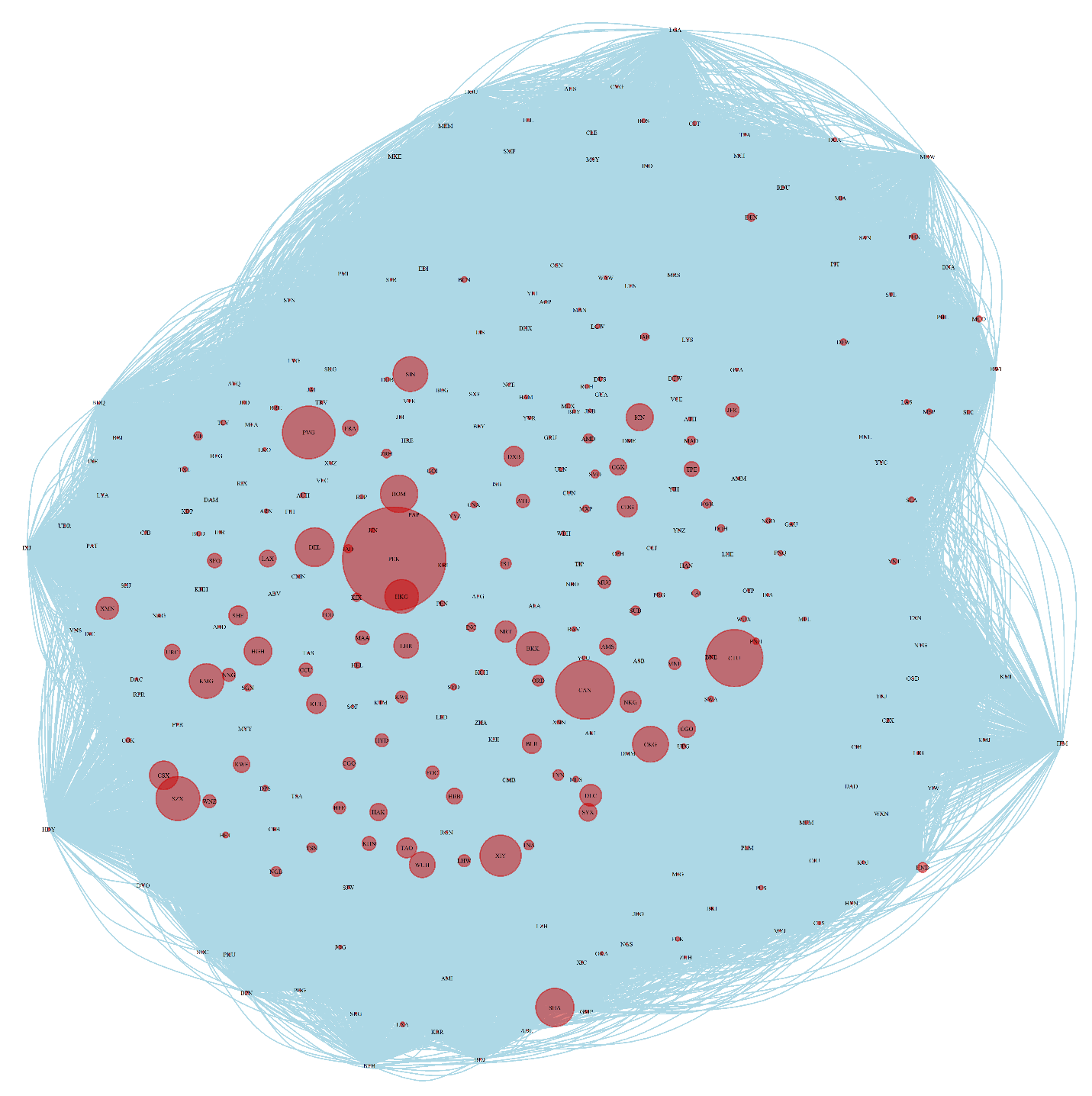


S3 Table 1. Variance metrics for the loadings of the principal components analysis of the network centrality metrics.

| A. Metric | PC1 | PC2 |
| --- | --- | --- |
| Standard deviation | 1.3443 | 0.43915 |
| Proportion of variance | 0.9036 | 0.09643 |
| Cumulative proportion of variance | 0.9036 | 1.0 |
| B. Life history trait | PC1 | PC2 |
| Strength | 0.7071068 | -0.7071068 |
| Eigenvector centrality | 0.7071068 | 0.7071068 |

S4 Figure 3. Alert-level yellow zones depict two-feature interfaces between mammalian and bird richness and each of the human population, livestock, and poultry densities separately based on the intersection of the 50^th^ percentile of each feature’s distribution. Cities of high network centrality (50^th^ percentile) based on global air travel and within 50 km of yellow interfaces are overlaid. The proportion of highly connected transportation hubs (50^th^ percentile of airport network centrality) within 50 km of each interface is presented with the airplane icon. Cities mapped twice have two high centrality airports.


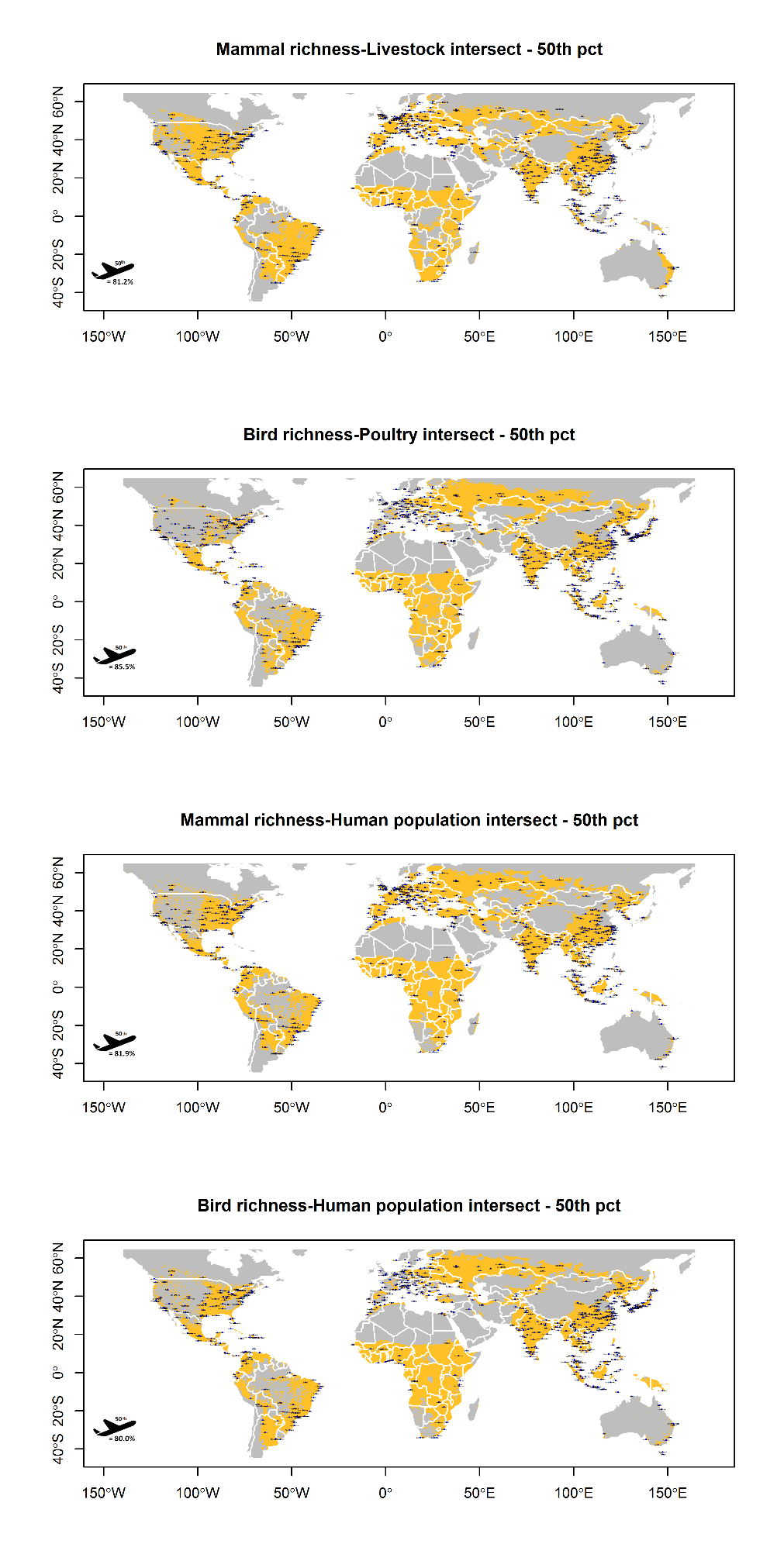


S5 Figure 4. Alert-level orange zones depict three-feature interfaces between mammalian and bird richness, human population density, and each of the livestock and poultry densities separately based on the intersection of the 50^th^ percentile of each feature’s distribution. Cities of high network centrality (50^th^ percentile) based on global air travel and within 50 km of orange interfaces are overlaid. The proportion of highly connected transportation hubs (50^th^ percentile of airport network centrality) within 50 km of each interface is presented with the airplane icon. Cities mapped twice have two high centrality airports.


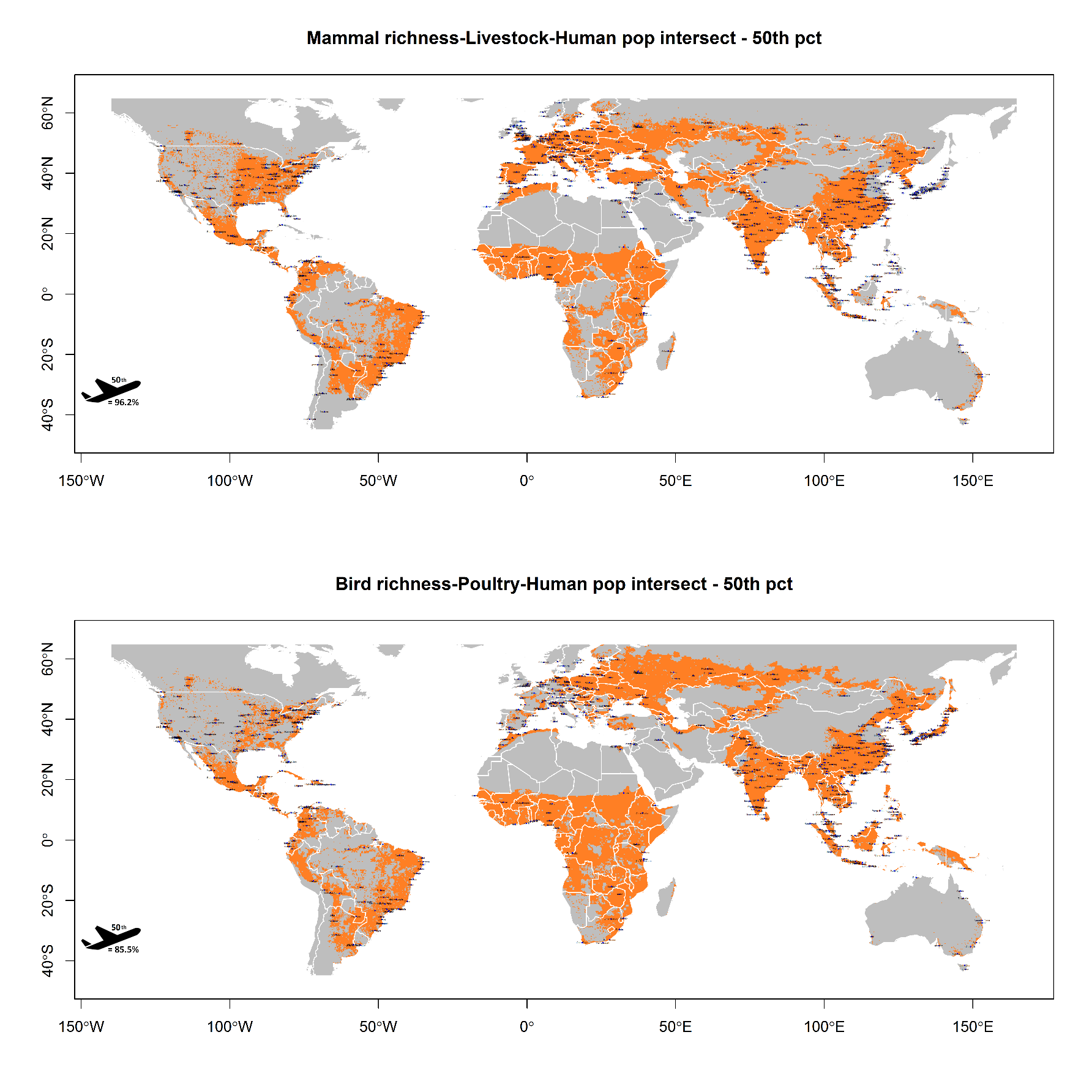


S6 Figure 5. Alert-level red zones depict the same animal-human interfaces as presented in alert-level orange, but extends the intersection of these interfaces with the 50^th^ percentile of infant mortality. Cities of high network centrality (50^th^ percentile) based on global air travel and within 50 km of red interfaces are overlaid. The proportion of highly connected transportation hubs (50^th^ percentile of airport network centrality) within 50 km of each interface is presented with the airplane icon. Cities mapped twice have two high centrality airports.


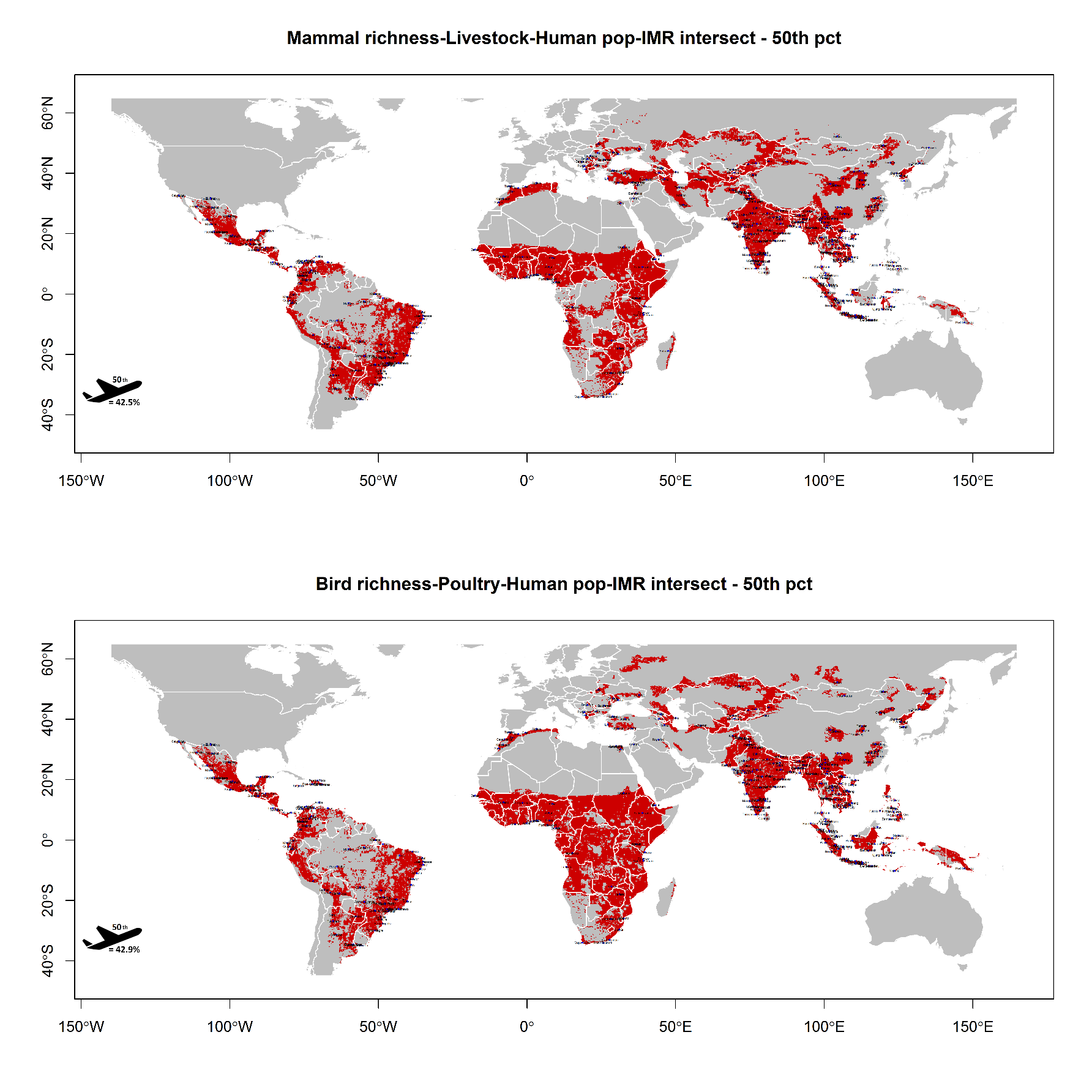

Supplement: Supplementary file 1 — Supplementary material [file mmc1.docx]
